# Socioeconomic disparities in endometrial cancer survival in Germany: a survival analysis using population-based cancer registry data

**DOI:** 10.1007/s00432-021-03908-9

**Published:** 2022-01-22

**Authors:** Ahmed Bedir, Semaw Ferede Abera, Dirk Vordermark, Daniel Medenwald

**Affiliations:** 1 Health Services Research Group, Department of Radiation Oncology, University Hospital Halle (Saale), Ernst-Grube-Str. 40, 06120 Halle (Saale), Germany; 2 Department of Radiation Oncology, University Hospital Halle (Saale), Ernst-Grube-Str. 40, 06120 Halle (Saale), Germany

**Keywords:** Endometrial cancer, Socioeconomic deprivation, Survival analysis

## Abstract

**Purpose:**

Area-based socioeconomic deprivation has been established as an important indicator of health and a potential predictor of survival. In this study, we aimed to measure the effect of socioeconomic inequality on endometrial cancer survival.

**Methods:**

Population-based data on patients diagnosed with endometrial cancer between 2004 and 2014 were obtained from the German Centre for Cancer Registry Data. Socioeconomic inequality was defined by the German Index of Socioeconomic Deprivation. We investigated the association of deprivation and overall survival through Kaplan–Meier curves and Cox proportional regression models.

**Results:**

A total of 21,602 women, with a mean age of 67.8 years, were included in our analysis. The observed 5-year overall survival time for endometrial cancer patients living in the most affluent districts (first quintile) was 78.6%. The overall survival rate decreased as the level of deprivation increased (77.2%, 73.9%, 76.1%, 74.7%, for patients in the second, third, fourth, and fifth quintile (most deprived patients), respectively). Cox regression models showed stage I patients living in the most deprived districts to have a higher hazard of overall mortality when compared to the cases living in the most affluent districts [Hazard ratio: 1.20; 95% Confidence interval (0.99–1.47)] after adjusting for age, tumor characteristics, and treatment.

**Conclusion:**

Our results indicate differences in endometrial cancer survival according to socioeconomic deprivation among stage I patients. Considering data limitations, future studies with access to individual-level patient information should be conducted to examine the underlying causes for the observed disparity in cancer survival.

**Supplementary Information:**

The online version contains supplementary material available at 10.1007/s00432-021-03908-9.

## Introduction

Endometrial cancer (EC) is the most commonly diagnosed gynecological cancer in Germany, with 12,356 new cases and 2444 deaths being reported in 2020 alone (Sung et al. 2021). According to the International Agency for Research on Cancer, the incidence of EC is projected to rise up to 5% within the next 10 years (Ferlay et al. 2020). While the 5-year relative survival rate is estimated to be about 78%, few studies have investigated potential regional differences concerning EC survival within Germany (Robert-Koch-Institut 2019).

In a recent study by Finke et al., the majority of cancer patients living in the most socioeconomically deprived municipalities were found to have significantly lower survival compared to the most affluent patients in Germany (Finke et al. 2021). These findings confirm the survival disparity reported in previous studies (Brenner et al. 1991; Jansen et al. 2014, 2020, 2021). In regard to EC, social deprivation could affect clinical outcomes on several levels from early pathogenesis to stage at diagnosis and treatment. Important risk factors such as obesity, comorbidities, and smoking are especially prevalent in deprived populations (Amant et al. 2005; Arem and Irwin 2013; Bouwman et al. 2015; Donkers et al. 2020; Dragano et al. 2007). Moreover, the availability and access to care could prove to be crucial to women diagnosed at later stages when a more complex treatment plan is required (Network 2021).

Considering the impact of area-based socioeconomic deprivation and how it is considered today as an important indicator of health (Diez Roux 2016; Marmot et al. 1987; Pickett and Pearl 2001), it is therefore, our aim to explore survival inequalities related to EC. Using data from German population-based cancer registries, we measured the association between area-based socioeconomic deprivation and endometrial cancer survival on the district level. Furthermore, we examined whether this association depended on the age at diagnosis, tumor characteristics, or the cancer therapy received.

## Materials and methods

### Data source and study population

This retrospective study is based on population-based cancer registry data from 8 out of 16 German federal states (Nordrhein-Westfalen, Hessen,^1^ Bayern, Brandenburg, Mecklenburg-Vorpommern, Sachsen, Sachsen-Anhalt, and Thüringen) covering a population of 49.9 million people (~ 59% of the total German population). The data was pooled and provided by the German Centre for Cancer Registry Data at the Robert Koch Institute (RKI) (https://doi.org/10.18444/5.03.01.0005.0015.0001) (Hiripi et al. 2012). The overall proportion of death certificate only (DCO) cases in the period 2004–2014 was calculated to ensure that the proportion in the included registries did not exceed the recommended 13% (Rossi et al. 2015) (Table 1).

**Table 1:** Description of the cancer registries and administrative districts included in our analysis, 2004–2014

Cancer registry	Population (Million in 2017)	% DCO Cases	Cases^a^	Mean GISD of included districts (SD)	Number of included districts
Nordrhein-Westfalen	17.91	3.1%	5339	0.62 ± 0.13	53
Hessen^b^	6.29	8.3%	635	0.51 ± 0.16	26
Bayern	13.14	3.8%	2892	0.50 ± 0.12	84
Brandenburg	2.53	1.1%	2129	0.80 ± 0.11	18
Mecklenburg-Vorpommern	1.61	2.0%	1134	0.87 ± 0.05	8
Sachsen	4.06	0.7%	4859	0.75 ± 0.08	13
Sachsen-Anhalt	2.18	2.8%	2073	0.88 ± 0.06	14
Thüringen	2.12	2.2%	2547	0.76 ± 0.10	23
Total	49.84	3.2%	21,602	0.63 ± 0.18	239

*DCO* death certificate only, *GISD* German Index of Socioeconomic Deprivation, *SD* standard deviation

^a^Final number of cases diagnosed with endometrial cancer, 2004–2014, after excluding DCO and autopsy-only cases

^b^Patients diagnosed in Darmstadt, Hessen before 2007 were not available in the respective cancer registry data

Women at the age of 18 years or older with a primary diagnosis of endometrial cancer (International Classification of Diseases for Oncology topography codes C541) diagnosed during 2004–2014 were included in this analyses. Follow-up as recorded in the registries ended in December 2014. Cases notified by autopsy only or by death certificate only (DCO) were excluded. Only complete cases were included in our analysis.

### Exposure and outcome

The exposure of interest was the socioeconomic deprivation level of the respective case, which was determined by the German Index of Socioeconomic Deprivation (GISD) (Kroll et al. 2017) allocated to the residential district of the case at the time of diagnosis.

The GISD is a composite index of three equally weighted socioeconomic domains: income, education and employment. The income dimension is based on the mean net household income, tax revenues, and debtor quotas within a given district. The educational component is defined by the proportions of employees in the district with (and without) a university degree, school dropouts without a degree, and school dropouts with the German “Abitur” or equivalent. Finally, the employment dimension is measured through the local unemployment rate, average gross wage of employees, and the labor force participation rate. The index ranges on a scale of zero to one, with zero representing the lowest level of socioeconomic deprivation (most affluent) and one representing the most socioeconomically deprived districts. The indices were then categorized into five quintiles [Q1 (least deprived)-Q5 (most deprived)].

**Fig. 1:**
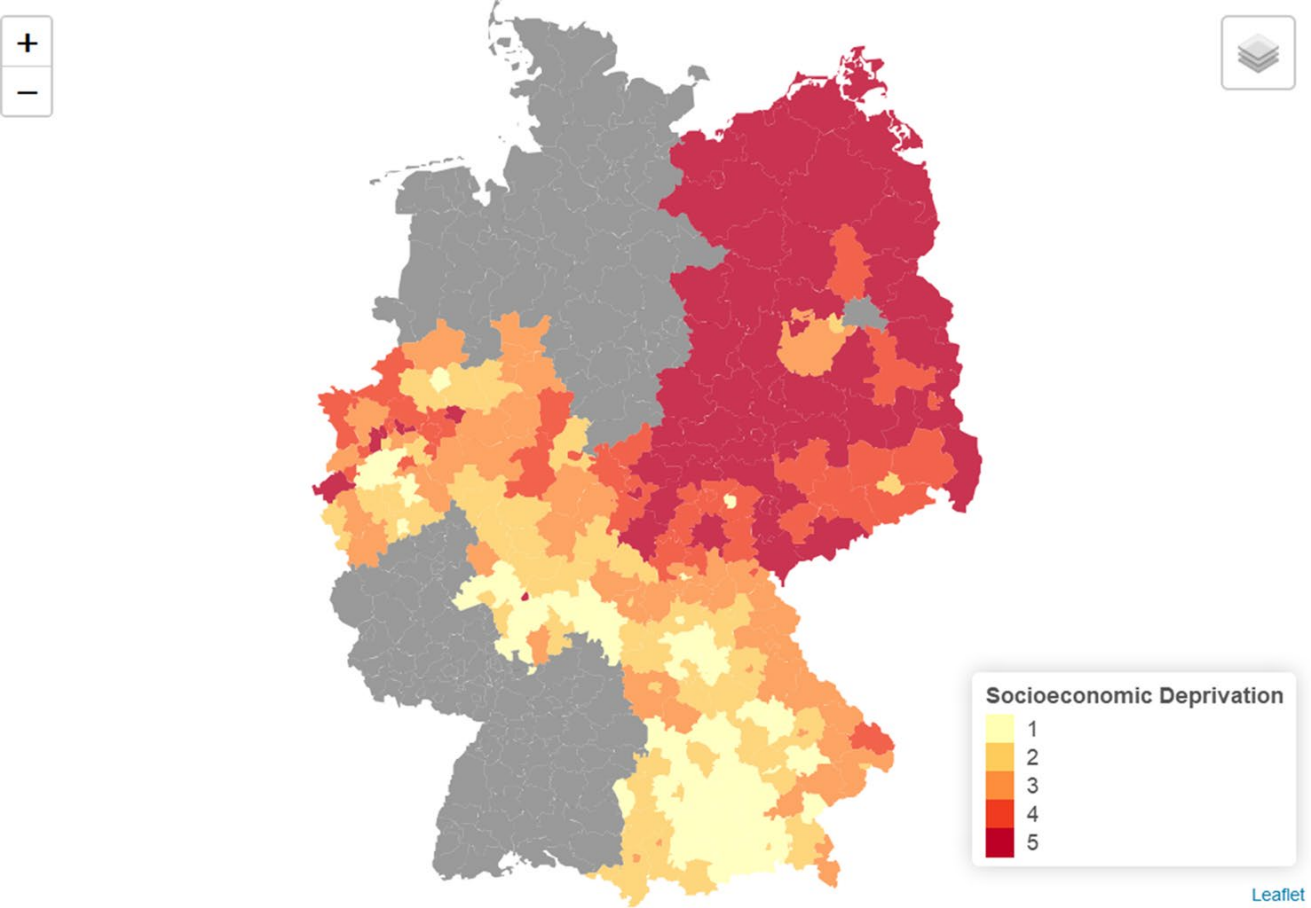
Map of Germany with districts included in the analysis, colored according to their mean level of socioeconomic deprivation over the study period, 2004–2014. Quintiles are listed in ascending order according to deprivation (quintile five = most deprived)

In the end, 239 districts out of 401 German districts were included in our study after being linked with the pooled registry dataset (Fig. 1).

The primary outcome measured was overall survival (OS). Overall survival was computed from date of cancer diagnosis to date of death from any cause. Vital status was ascertained using death certificates and information from the registration offices. Patients lost to follow-up before death or still alive at the last vital status assessment were right-censored at the date of the last vital status assessment or at the censor date (December 2014), whichever came first.

### Covariates

The pooled dataset contained information on grading and histology, TNM (tumor–node–metastasis) stage, cause and date of death, date of birth and date of diagnosis, and treatment. We categorized stage at diagnosis into four groups based on the TNM cancer staging system (Edge et al. 2010). We also classified endometrial carcinoma according to its two subtypes: I (low-grade) and II (high-grade). Type I included endometrioid adenocarcinoma and its variants: villoglandular, secretory, with ciliated cells, adenocarcinoma with squamous differentiation, and other unspecified adenocarinoma variants (histology codes 8380, 8382, 8383, 8480–8482, 8210, 8140, 8560, 8570). Type II histologies included serous, clear cell, mixed cell, small cell, and squamous cell adenocarcinomas (codes 8440, 8441, 8460, 8461, 8310, 8323, 8041, 8070, 8071, 8076) (Amant et al. 2005). Furthermore, we considered type I cases with grade 3 or worse, as type II cases.

Information on treatment was available as dichotomous variables (surgery yes/no, radiotherapy yes/no, chemotherapy yes/no). Details on administered radiation doses, specific chemotherapy treatment, or date of treatment were not available.

### Statistical analysis

Demographic and clinical characteristics according to deprivation quintiles were described using common descriptive statistics. The observable 5- and 10-year overall survival rates (OS) for each quintile was calculated and visualized by the Kaplan–Meier estimates and curves. Multivariate analysis was performed using the Cox proportional hazards model to investigate the association between area-based deprivation and survival. The hazard ratio (HR) and 95% confidence interval (CI) were reported using the most affluent quintile (Q1) as the reference group. The potential impact contributed by our covariates to the survival disparity between the quintiles was assessed by entering these factors sequentially into our cox proportional hazards models. The base model included adjustment for age and year of diagnosis. We further adjusted for subtype and grading, tumor stage, and treatment in models 2, 3, and 4 respectively. In an additional fifth model, we adjusted for cancer registry. All analyses were conducted in R statistical software version 3.2.3 (Team 2013).

### Sensitivity analysis

To assess the robustness of our findings, we explored potential bias arising from missing stage and treatment information. We assumed missing stage information to be missing at random (MAR). As a result, we used multiple imputation using chained equations (implemented in the R package “mice”) to impute missing stage (van Buuren and Groothuis-Oudshoorn 2010). Our imputation model included all variables from our complete cases dataset. Based on five imputed datasets, we repeated our analysis to include previously excluded patients.

On the other hand, we found that the process of recording treatment information varied across the German cancer registries. The included registries from former West Germany (Nordrhein-Westfalen, Hessen, and Bayern) documented treatment as “received”, “not received”, or truly “unknown” (missing). Cases with missing treatment information within these registries were excluded from the main analysis. In the former East German states (Brandenburg, Mecklenburg-Vorpommern, Sachsen, Sachsen-Anhalt, and Thüringen) however, all patients are initially recorded as having received “no treatment” until the notifying institution provides information on the treatment procedure performed, whereupon the respective case’s status changes from treatment “not received” to “received”. Therefore, these registries did not include missing treatment information since there was no differentiation between a certain procedure being truly “not received” or if it was “missing” for that matter. In a sensitivity analysis, we recoded cases (from former West German registries) with missing treatment information as “not treated” and repeated our cox regression models.

## Results

### Descriptive

**Fig. 2:**
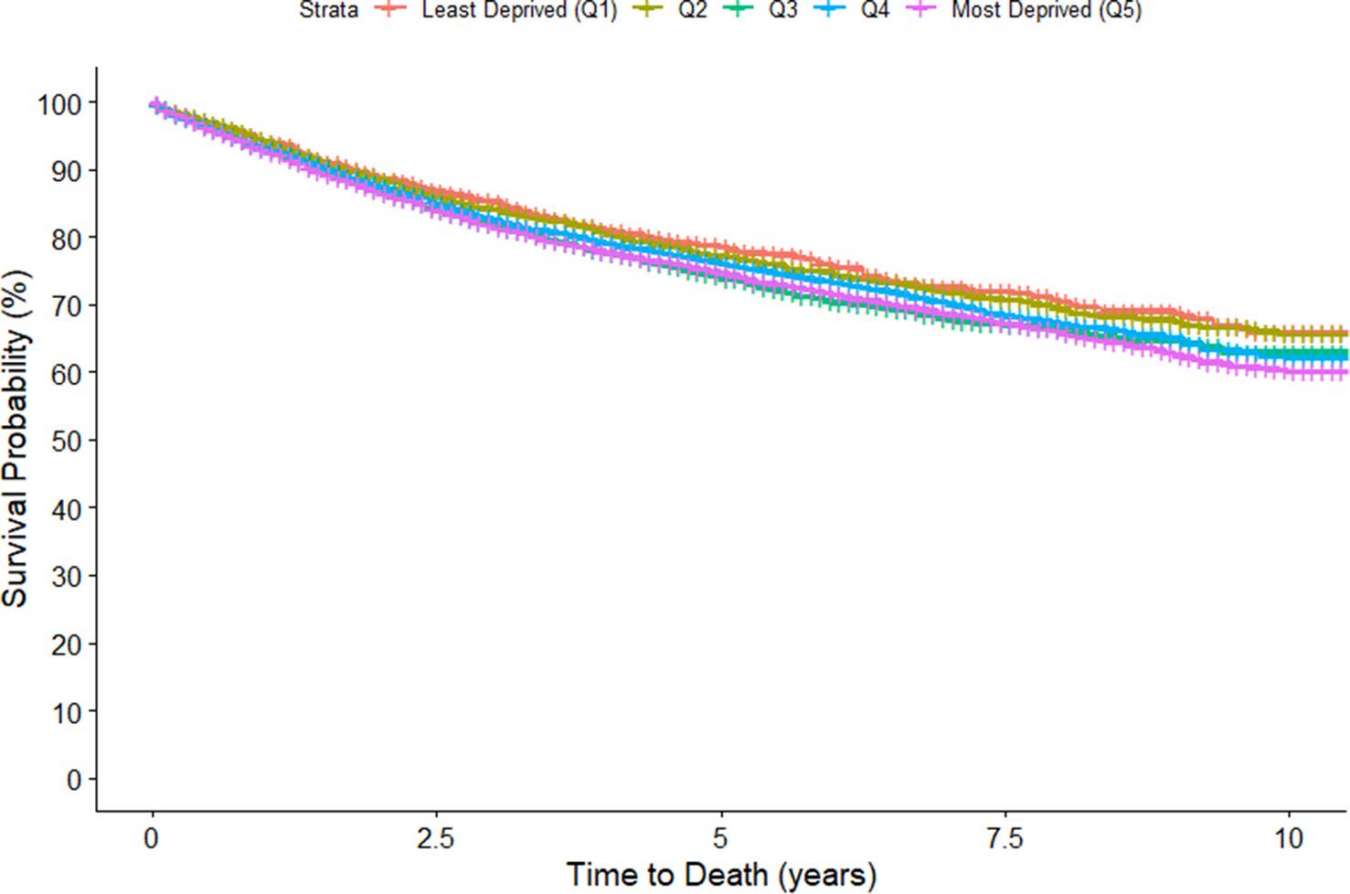
Kaplan Meier Curves comparing 10 year overall survival of endometrial cancer patients diagnosed 2004–2014 according to deprivation quintiles

In total, 21,602 cases diagnosed with endometrial cancer between 2004 and 2014 were included in our analysis (Table 2). Of the patients living in the most deprived districts, 68.9% survived up to the end of follow-up compared to 71.3% of the patients living in the least deprived districts at the time of diagnosis. The mean age at diagnosis for all patients was 67.8 ± 11.2 years (range 24–104) with the patients living in the most deprived districts being the oldest among the quintiles (68.3 ± 11.0). With regard to subtypes and tumor grading distribution, patients living in the more affluent districts were more likely to be diagnosed with the high-grade variant of EC. The vast majority of the cases were diagnosed at stage I across all the groups. The proportions of patients receiving treatment (radiotherapy, chemotherapy, or surgery) seemed to drop as the deprivation level of the district increased. The observed 5-year overall survival (OS) time was the highest for Q1 patients 78.6% (95% CI 76.3–80.9) and lowest for patients in Q3 (73.1%, 95% CI 72.1–76.0) and Q5 (74.7%, 95% CI 73.6–75.8) (Table 3, Fig. 2). The 10-year OS time followed a similar pattern with patients in Q5 showing the worst survival (60.2%, 95% CI 58.5–61.9) and Q1 having the best 10-year OS (66.0%, 95% CI 62.3–69.9).

**Table 2:** Characteristics of patients diagnosed with endometrial cancer 2004–2014 according to socioeconomic deprivation quintiles

	All patients	Deprivation level				
		Least deprived	2	3	4	Most deprived
Number of patients	21,602	1685	3146	2908	5604	8259
Alive at end of follow-up (%)	14,985 (69.4)	1202 (71.3)	2213 (70.3)	1967 (67.6)	3915 (69.9)	5688 (68.9)
Mean age at diagnosis (SD)	67.8 (11.2)	66.7 (11.2)	67.0 (11.5)	66.9 (11.4)	68.2 (11.0)	68.3 (11.0)
Period of diagnosis (%)						
2004–2008	9315 (43.1)	675 (40.1)	1382 (43.9)	1108 (38.1)	2435 (43.5)	3715 (45.0)
2009–2013	12,287 (56.9)	1010 (59.9)	1764 (56.1)	1800 (61.9)	3169 (56.5)	4544 (55.0)
Type (%)						
Low grade	17,225 (79.7)	1288 (76.4)	2472 (78.6)	2245 (77.2)	4515 (80.6)	6705 (81.2)
High grade	4377 (20.3)	397 (23.6)	674 (21.4)	663 (22.8)	1089 (19.4)	1554 (18.8)
Grade (%)						
I	8248 (38.2)	552 (32.8)	1094 (34.8)	1047 (36.0)	2109 (37.6)	3446 (41.7)
II	9175 (42.5)	756 (44.9)	1403 (44.6)	1221 (42.0)	2465 (44.0)	3330 (40.3)
III	4179 (19.3)	377 (22.4)	649 (20.6)	640 (22.0)	1030 (18.4)	1483 (18.0)
Stage at diagnosis (%)						
I	11,699 (54.2)	852 (50.6)	1602 (50.9)	1330 (45.7)	3264 (58.2)	4651 (56.3)
II	1244 (5.8)	109 (6.5)	164 (5.2)	152 (5.2)	310 (5.5)	509 (6.2)
III	1564 (7.2)	154 (9.1)	270 (8.6)	204 (7.0)	375 (6.7)	561 (6.8)
IV	530 (2.5)	58 (3.4)	84 (2.7)	70 (2.4)	125 (2.2)	193 (2.7)
Missing	6565 (30.4)	512 (30.4)	1026 (32.6)	1152 (39.6)	1530 (27.3)	2345 (28.4)
Treatment (%)						
Radiotherapy	8832 (40.9)	691 (41.0)	1263 (40.1)	1096 (37.7)	2393 (42.7)	3389 (41.0)
Chemotherapy	1181 (5.5)	163 (9.7)	252 (8.0)	206 (7.1)	211 (3.8)	349 (4.2)
Surgery	20,438 (94.6)	1644 (97.6)	3055 (97.1)	2702 (92.9)	5393 (96.2)	7666 (92.8)

*SD* standard deviation

**Table 3:** Kaplan–Meier survival estimates according to deprivation levels of patients diagnosed with endometrial cancer in Germany, 2004–2014

Deprivation quintiles	Kaplan–Meier estimated overall survival (unadjusted) (95% CI)	
All stages	5-year	10-year^a^
Quintile 1	78.6 (76.3–80.9)	66.0 (62.3–69.9)
Quintile 2	77.2 (75.5–78.9)	65.8 (63.1–68.5)
Quintile 3	73.9 (72.1–76.0)	63.0 (60.0–66.1)
Quintile 4	76.1 (74.9–77.4)	62.2 (60.0–64.4)
Quintile 5	74.7 (73.6–75.8)	60.2 (58.5–61.9)

*CI* confidence interval

^a^Patients diagnosed in Darmstadt, Hessen before 2007 were not available in the respective cancer registry data, therefore were not included in the 10-year survival analysis

### Cox models

Our base cox regression model for the total population did not show an association between overall mortality and socioeconomic deprivation. Our stratified analysis on the other hand, consistently showed a higher hazard of overall mortality for the stage I patients living in the more deprived districts (especially Q3 and Q5). After including patient and tumor characteristics in addition to treatment received information to our model, Q5 showed the highest hazard of overall mortality when compared to our reference group (Q1) [HR 1.20, 95% CI (0.99–1.47)] (Table 4). Adjusting for registry did not alter our estimates. No association was seen in patients diagnosed at later stages.

### Sensitivity analysis

Twenty eight percent of patients in the Q5 had missing stage information in comparison to 30.4% in Q1 (and 39.6% in Q3). After using the available information in our data to impute five complete datasets, slightly more patients in the deprived districts appeared to survive at the end of follow-up when compared to the affluent group. In contrast to the original data, a smaller proportion of patients in Q1 and Q2 were diagnosed during the later period of 2009–2013. The distribution of stage at diagnosis, tumor grading, treatment, and the patients’ characteristics, did not change across the groups when compared to our original dataset (Online Appendix 1). After repeating the regression analysis, an association between deprivation and overall mortality was more evident in the total population even after adjusting for tumor characteristics and treatment received variables (Online Appendix 1). This association was replicated among stage I patients. Unlike the original analysis, the imputed dataset showed patients diagnosed at stage II and III, to have also been affected by the socioeconomic-based disparity in survival.

**Table 4:** Cox proportional hazards model survival estimates according to deprivation levels of patients diagnosed with endometrial cancer in Germany, 2004–2014

	*N* of Events	Hazard ratios (95% CI)				
		Model 1	Model 2	Model 3	Model 4	Model 5
All Stages	3038					
Q1		1.00 (ref)	1.00 (ref)	1.00 (ref)	1.00 (ref)	1.00 (ref)
Q2		0.91 (0.78–1.06)	0.89 (0.76–1.04)	0.92 (0.78–1.07)	0.90 (0.77–1.05)	0.90 (0.77–1.04)
Q3		1.03 (0.88–1.22)	1.00 (0.85–1.18)	1.03 (0.87–1.21)	1.00 (0.86–1.18)	1.02 (0.86–1.20)
Q4		0.85 (0.73–0.97)	0.87 (0.75–1.00)	0.95 (0.83–1.10)	0.94 (0.81–1.09)	0.92 (0.79–1.07)
Q5		0.94 (0.82–1.08)	0.98 (0.85–1.12)	1.02 (0.89–1.17)	1.01 (0.88–1.16)	0.98 (0.84–1.14)
Stage I	1701					
Q1		1.00 (ref)	1.00 (ref)		1.00 (ref)	1.00 (ref)
Q2		1.01 (0.81–1.27)	0.99 (0.78–1.24)		0.98 (0.78–1.23)	0.98 (0.78–1.23)
Q3		1.27 (1.00–1.60)	1.22 (0.96–1.54)		1.19 (0.94–1.50)	1.18 (0.93–1.50)
Q4		1.06 (0.86–1.30)	1.05 (0.85–1.29)		1.05 (0.85–1.29)	1.06 (0.85–1.32)
Q5		1.19 (0.98–1.46)	1.20 (0.99–1.47)		1.20 (0.99–1.47)	1.21 (0.97–1.50)
Stage II	339					
Q1		1.00 (ref)	1.00 (ref)		1.00 (ref)	1.00 (ref)
Q2		0.62 (0.37–1.01)	0.60 (0.37–1.00)		0.59 (0.36–0.99)	0.60 (0.36–0.98)
Q3		0.88 (0.55–1.41)	0.90 (0.56–1.44)		0.89 (0.55–1.43)	0.93 (0.58–1.51)
Q4		0.88 (0.58–1.35)	0.89 (0.59–1.36)		0.88 (0.58–1.34)	0.81 (0.51–1.26)
Q5		0.87 (0.58–1.30)	0.87 (0.58–1.30)		0.86 (0.57–1.28)	0.76 (0.48–1.19)
Stage III	659					
Q1		1.00 (ref)	1.00 (ref)		1.00 (ref)	1.00 (ref)
Q2		1.01 (0.75–1.37)	1.05 (0.77–1.42)		1.00 (0.73–1.36)	1.01 (0.74–1.37)
Q3		0.93 (0.68–1.28)	1.03 (0.75–1.43)		0.97 (0.70–1.35)	1.00 (0.72–1.40)
Q4		0.99 (0.74–1.32)	1.07 (0.80–1.43)		1.01 (0.76–1.36)	0.97 (0.72–1.32)
Q5		0.94 (0.71–1.24)	1.00 (0.76–1.31)		0.97 (0.73–1.28)	0.91 (0.67–1.23)
Stage IV	339					
Q1		1.00 (ref)	1.00 (ref)		1.00 (ref)	1.00 (ref)
Q2		0.83 (0.55–1.22)	0.80 (0.54–1.20)		0.78 (0.52–1.18)	0.79 (0.53–1.18)
Q3		0.81 (0.53–1.24)	0.76 (0.50–1.17)		0.71 (0.46–1.09)	0.73 (0.47–1.12)
Q4		0.64 (0.44–0.94)	0.61 (0.43–0.93)		0.54 (0.37–0.83)	0.51 (0.32–0.80)
Q5		0.75 (0.53–1.06)	0.73 (0.52–1.04)		0.67 (0.47–0.95)	0.61 (0.40–0.92)

Model 1: Adjusted for age and year of diagnosis. Model 2: Same as Model 1 plus Grade and Type. Model 3: Same as Model 2 plus stage Model 4: Same as Model 3 plus treatment. Stratified analysis: Same as Model 2 plus treatment, Model 5: Same as Model 4 plus registry. Stratified analysis: Same as Model 2 plus treatment and registry

*Q* quintiles, *CI* confidence intervals, *ref* reference group

Reincluding cases with missing treatment information from the former West German cancer registries in our sensitivity analysis almost doubled the number of cases in Q1–3. This increase however, was also accompanied by an increase in the proportion of patients with missing stage at diagnosis. Overall the sensitivity analysis was conducted using 17,221 complete cases (1836 in Q1) compared to 15,037 patients used in the original analysis (1173 in Q1). The results from the cox regression models replicated the main results from the original analysis (Online Appendix 2).

## Discussion

In this study, we found differences in endometrial cancer survival according to district-level socioeconomic deprivation. The regression models highlighted the association between deprivation level and overall survival in stage I endometrial cancer patients, with better survival for the patients living in the least deprived districts. This association remained after adjusting for patient and tumor characteristics and the treatment received. No effect was detected however, in patients diagnosed at later stages. This could be partly explained by the relatively small number of patients diagnosed at those stages across the five quintiles. Our sensitivity analysis, while confirming our main findings, revealed that missing stage information could have also played a role in influencing the results.

When comparing our findings to studies performed in other countries that offer a publicly accessible universal health care system, similar to the system present in Germany, we found the results to be somewhat comparable. Patients from lower socioeconomic groups in North West of England were found to have a 53% (adjusted HR = 1.53, 95% CI 0.77–3.04) increase in cancer‐specific mortality when compared with affluent patients (Njoku et al. 2020). Another study conducted in Denmark during 1994–2003 concluded that increased excess mortality rates from endometrial cancer were associated with low educational level, mainly during the first year after diagnosis (Jensen et al. 2008).

In Germany however, as of the writing of this paper, we were unable to find studies that dealt with this topic. Jansen et al. (2021) measured the 5-year age standardized relative survival of women diagnosed with Corpus Uteri cancer, among other cancer sites, during the period between 2013 and 2017. The study was based on 200 administrative German districts representing approximately 39% of the entire population. The authors found no significant differences in terms of net survival between the most deprived (80.3%) and the most affluent patients (81.6%). The relative excess risk (RER) reported showed the most deprived patients to have an increased RER of death (adjusted for age at diagnosis RER: 1.11 95% CI (0.99–1.25)) compared to the least deprived (Jansen et al. 2021). These finding were similar to those reported by Finke et al. (2021). Finke et al. reported RERs adjusted for age and stage at diagnosis for patients diagnosed during the period of 2012–2014. The most deprived patients again showed an increased RER of death (1.08 95% CI (0.91–1.30) compared to the least deprived. These studies however, did not adjust for treatment information. It is also worth noting, that Corpus Uteri cancer (ICD-10 C54) encompasses tumors that arise in both the endometrium and myometrium, albeit 90% of uterine cancers originate from the endometrium.

The findings that cases are diagnosed at an early stage where treatment is less complex are especially prone to the effect of socioeconomic status might give reason to the argument that treatment is not the main contributor to these effects. Behavioral factors such as obesity affecting also non-cancer mortality might play the dominant role.

### Strengths and limitations

The main strength of this study is its attempt to fill the current gap in literature concerning the association between socioeconomic deprivation and endometrial cancer survival. Despite the recent growing interest in the effect of deprivation on cancer survival in general, as evident in newly published studies (Bedir et al. 2021; Finke et al. 2020; Jansen et al. 2020; Kuznetsov et al. 2011), our study is the first to focus on endometrial cancer in Germany. Our findings could be considered nationally representative, since they are based on eight cancer registries representing almost 50 million people from 239 German districts (out of 401) from both former East and West German states. Another strength of this study is that our analysis included information on treatment, which was not the case in previous studies. Our data also included all stages and grouped all known histological variants of EC into the respective subtypes. By stratifying our analysis according to stage, we ruled out the probability that differential stage at diagnosis could have had an effect on survival. According to the literature and as supported by the baseline characteristics of our sample, the majority of the EC patients are usually diagnosed at stage I (Amant et al. 2005). Fewer cases were diagnosed at later stages in our dataset, thus producing no effect in the cox models. When we imputed missing stage information, the distribution of stage at diagnosis remained relatively the same as the original dataset, but with the increased number of cases, our cox models revealed a higher hazard of overall mortality in patients diagnosed at stages II and III and are living in the more deprived quintiles at the time of diagnosis.

In contrast to Jansen et al., we used the GISD, instead of the German Index of Multiple Deprivation (GIMD), as a measure of deprivation. As explained earlier, the GISD is based solely on three classical dimensions of socioeconomic inequality (education, income, and employment) and is publicly available. This helps make the analysis reproducible and the results easier to interpret.

Nevertheless, the GISD has its limitations. It is an area-based index and is not based on, for example, the individual’s income or level of education. This could lead to the misclassification of patients by grouping individuals from a higher socioeconomic position into the most deprived socioeconomic quintile because they live in a district where the majority of its residents have a lower socioeconomic status. We were unable to measure the magnitude of this potential misclassification or its effect on our results, since individual-level information on socioeconomic position was not available in our dataset. However, since GISD covers a wide range of socioeconomic indicators, we believe it to be an accurate measurement for deprivation since it has been used in previous research (Hoebel et al. 2018; Moissl et al. 2020; Rommel et al. 2018).

Retrospective studies based on cancer registry data have several limitations. German registries do not systematically collect data on comorbidities or lifestyle-related EC risk factors such as smoking, unhealthy diets, physical activity, and obesity which have been proven to be directly related to socioeconomic status as well as cancer survival in general (Sarfati et al. 2016; Søgaard et al. 2013).

Another limitation of our study is the varied process of recording treatment information by different German cancer registries. The results of our sensitivity analysis did not differ from the main analysis; however, a standardized definition of “missing” across the cancer registries could help provide a more accurate insight on the effect of treatment on survival.

Furthermore, the registries do not contain data on the process of treatment decision, when was the treatment performed, or if the patient was publicly or privately insured. These unmeasured confounders could have led to the overestimation of the effect of socioeconomic deprivation.

## Conclusion

Our results indicated differences in endometrial cancer survival according to socioeconomic deprivation among patients diagnosed at stage I. Future studies, with access to individual-level patient information, could take advantage of helpful tools like directed acyclic graphs (DAGs) in visualizing and explaining the underlying mechanism by which a complex factor, like area-based socioeconomic deprivation, would affect cancer survival.

## Supplementary Information

Below is the link to the electronic supplementary material.

Supplementary file1 (PDF 319 KB)

Supplementary file2 (PDF 277 KB)

Supplementary file3 (PDF 300 KB)

## Data Availability

This study was based on the German national cancer registry data. The authors do not own these data and hence are not permitted to share them in the original form (only in aggregate form, eg, publications). Data were provided by the Robert Koch Institute (RKI) (https://doi.org/10.18444/5.03.01.0005.0015.0001).

## Footnotes

1 Information on cases from Darmstadt, a city in the state of Hessen with a population of 159,207 (2019), was not available in the Hessen cancer registry data prior to 2007.
