# Supplementary material for: Socioeconomic disparities in endometrial cancer survival in Germany: a survival analysis using population-based cancer registry data
Source: J Cancer Res Clin Oncol. 2022 Jan 22;148(5):1087–95. doi: 10.1007/s00432-021-03908-9 (PMC9015991; doi:10.1007/s00432-021-03908-9)
Supplement: Supplementary file 1 — Supplementary file1 (PDF 319 KB) [file 432_2021_3908_MOESM1_ESM.pdf]

Socioeconomic disparities in Endometrial Cancer survival in Germany: A survival analysis using population-based cancer registry data.

Bedir, Ahmed[1]; Abera, Semaw Ferede[1]; Vordermark, Dirk[1,2]; Medenwald, Daniel[1,2]

1. Department of Radiation Oncology, Health Services Research Group, University Hospital Halle (Saale), Ernst-Grube-Str. 40, 06120, Halle (Saale), Germany.
2. Department of Radiation Oncology, University Hospital Halle (Saale), Ernst-Grube-Str. 40, 06120, Halle (Saale), Germany.

Acknowledgments: None.

Address for correspondence:
Daniel Medenwald
Department of Radiation Oncology, University Hospital Halle (Saale),
Ernst-Grube-Str. 40, 06120, Halle (Saale), Germany.
Telephone no: +49-345-557-3453/4027
Email: Daniel.Medenwald@uk-halle.de

Appendix 1: Missing Stage information: Sensitivity Analysis

Table 1. Characteristics of patients diagnosed with endometrial cancer 2004-2014 according to socioeconomic deprivation quintiles based on five imputed datasets .

		Deprivation Level				
	All patients	**Least Deprived**	**2**	**3**	**4**	**Most Deprived**
Number of patients	101002	7712	14246	12638	27025	39381
Alive at end of follow-up (%)	65388 (64.7)	4962 (64.3)	9024 (63.3)	7446 (58.9)	18120 (67.0)	25836 (65.6)
Mean age at diagnosis **(SD)**	68.1 (11.0)	67.1 (11.1)	67.6 (11.3)	67.4 (11.3)	68.4 (10.9)	68.5 (10.9)
**Period of Diagnosis (%)**						
2004-2008	43620 (43.2)	3435 (44.5)	6569 (46.1)	4915 (38.9)	11556 (42.8)	17145 (43.5)
2009-2013	57382 (56.8)	4277 (55.5)	7677 (53.9)	7723 (61.1)	15469 (57.2)	22236 (56.5)
**Type (%)**						
Low grade	78376 (77.6)	5660 (73.4)	10856 (76.2)	9314 (73.7)	21268 (78.7)	31278 (79.4)
High grade	22626 (22.4)	2052 (26.6)	3390 (23.8)	3324 (26.3)	5757 (21.3)	8103 (20.6)
**Grade (%)**						
I	35887 (35.5)	2280 (29.6)	4310 (30.3)	4149 (32.8)	9581 (35.5)	15567 (39.5)
II	43460 (43.0)	3473 (45.0)	6659 (46.7)	5262 (41.6)	11970 (44.3)	16096 (40.9)
III	21655 (21.4)	1959 (25.4)	3277 (23.0)	3227 (25.5)	5474 (20.3)	7718 (19.6)
**Stage at Diagnosis (%)**						
I	75848 (75.1)	5472 (70.8)	10438 (73.3)	9107 (72.1)	20962 (77.6)	29884 (75.9)
II	8741 (8.7)	747 (9.7)	1142 (8.0)	1151 (9.1)	2136 (7.9)	3565 (9.1)
III	12001 (11.9)	1076 (14.0)	1979 (13.9)	1736 (13.7)	2892 (10.7)	4318 (11.0)
IV	4412 (4.4)	432 (5.6)	687 (4.8)	644 (5.1)	1035 (3.8)	1614 (4.1)
**Treatment (%)**						
Radiotherapy	44202 (43.8)	3542 (45.9)	6305 (44.3)	5304 (42.0)	12087 (44.7)	16964 (43.1)
Chemotherapy	6091 (6.0)	833 (10.8)	1281 (9.0)	1054 (8.3)	1141 (4.2)	1782 (4.5)
Surgery	96218 (95.3)	7564 (98.1)	13923 (97.7)	12123 (95.9)	25715 (95.2)	36893 (93.7)

Abbreviations: SD= Standard Deviation

Table 2: Cox proportional hazards model survival estimates according to deprivation levels of patients diagnosed with endometrial cancer in Germany, 2004–2014 based on five imputed datasets.

	N of Events	Hazard Ratios (95%CI)				
		Model 1	Model 2	Model 3	Model 4	Model 5
**All Stages**	28148					
Q1		1.00 (ref)	1.00 (ref)	1.00 (ref)	1.00 (ref)	1.00 (ref)
Q2		1.00 (0.95-1.06)	1.01 (0.96-1.07)	1.05 (1.00-1.11)	1.04 (0.98-1.10)	1.04 (0.98-1.10)
Q3		1.26 (1.19-1.33)	1.25 (1.18-1.31)	1.27 (1.20-1.32)	1.25 (1.18-1.32)	1.22 (1.16-1.30)
Q4		1.00 (0.95-1.05)	1.04 (1.00-1.10)	1.14 (1.08-1.20)	1.11 (1.05-1.17)	1.14 (1.09-1.21)
Q5		1.10 (1.05-1.15)	1.15 (1.10-1.21)	1.20 (1.15-1.25)	1.15 (1.08-1.21)	1.20 (1.14-1.26)
**Stage I**	15368					
Q1		1.00 (ref)	1.00 (ref)		1.00 (ref)	1.00 (ref)
Q2		1.09 (1.00-1.18)	1.08 (1.00-1.17)		1.08 (0.99-1.16)	1.08 (0.99-1.16)
Q3		1.49 (1.36-1.62)	1.44 (1.33-1.56)		1.40 (1.29-1.52)	1.37 (1.27-1.49)
Q4		1.19 (1.11-1.28)	1.20 (1.12-1.29)		1.18 (1.10-1.25)	1.25 (1.16-1.35)
Q5		1.31 (1.24-1.43)	1.34 (1.26-1.45)		1.31 (1.23-1.41)	1.39 (1.29-1.50)
**Stage II**	3246					
Q1		1.00 (ref)	1.00 (ref)		1.00 (ref)	1.00 (ref)
Q2		0.80 (0.67-0.94)	0.81 (0.69-0.96)		0.80 (0.67-0.94)	0.80 (0.67-0.94)
Q3		1.11 (0.94-1.30)	1.13 (0.97-1.33)		1.14 (0.97-1.33)	1.13 (0.96-1.32)
Q4		1.02 (0.88-1.18)	1.05 (0.91-1.21)		1.04 (0.90-1.20)	1.06 (0.91-1.23)
Q5		1.04 (0.90-1.19)	1.06 (0.92-1.21)		1.01 (0.88-1.16)	1.03 (0.89-1.20)
**Stage III**	6343					
Q1		1.00 (ref)	1.00 (ref)		1.00 (ref)	1.00 (ref)
Q2		1.11 (1.00-1.24)	1.16 (1.00-1.24)		1.10 (0.99-1.22)	1.10 (0.99-1.22)
Q3		1.15 (1.03-1.29)	1.25 (1.12-1.40)		1.16 (1.04-1.29)	1.18 (1.05-1.31)
Q4		1.16 (1.05-1.28)	1.25 (1.13-1.39)		1.13 (1.02-1.25)	1.11 (1.00-1.23)
Q5		1.15 (1.04-1.26)	1.22 (1.11-1.35)		1.09 (0.99-1.20)	1.05 (0.95-1.17)
**Stage IV**	3191					
Q1		1.00 (ref)	1.00 (ref)		1.00 (ref)	1.00 (ref)
Q2		0.96 (0.83-1.10)	0.96 (0.84-1.11)		0.98 (0.85-1.12)	0.98 (0.85-1.13)
Q3		0.94 (0.81-1.08)	0.92 (0.79-1.06)		0.89 (0.77-1.02)	0.89 (0.77-1.03)
Q4		0.83 (0.72-0.95)	0.84 (0.73-0.96)		0.77 (0.67-0.88)	0.75 (0.65-0.87)
Q5		0.91 (0.80-1.03)	0.91 (0.80-1.02)		0.83 (0.74-0.94)	0.81 (0.70-0.93)

Model 1: Adjusted for age and year of diagnosis. **Model 2**: Same as Model 1 plus Grade and Type. **Model 3**: Same as Model 2 plus stage Model 4: Same as Model 3 plus treatment. Stratified analysis: Same as Model 2 plus treatment, Model 5: Same as Model 4 plus registry. Stratified analysis: Same as Model 2 plus treatment and registry

Abbreviations: Q= Quintiles, CI= Confidence Interval.
