# Supplementary material for: Socioeconomic disparities in endometrial cancer survival in Germany: a survival analysis using population-based cancer registry data
Source: J Cancer Res Clin Oncol. 2022 Jan 22;148(5):1087–95. doi: 10.1007/s00432-021-03908-9 (PMC9015991; doi:10.1007/s00432-021-03908-9)
Supplement: Supplementary file 2 — Supplementary file2 (PDF 277 KB) [file 432_2021_3908_MOESM2_ESM.pdf]

Socioeconomic disparities in Endometrial Cancer survival in Germany: A survival analysis using population-based cancer registry data.

Bedir, Ahmed[1]; Abera, Semaw Ferede[1]; Vordermark, Dirk[1,2]; Medenwald, Daniel[1,2]

1. Department of Radiation Oncology, Health Services Research Group, University Hospital Halle (Saale), Ernst-Grube-Str. 40, 06120, Halle (Saale), Germany.
2. Department of Radiation Oncology, University Hospital Halle (Saale), Ernst-Grube-Str. 40, 06120, Halle (Saale), Germany.

Acknowledgments: None.

Address for correspondence:
Daniel Medenwald
Department of Radiation Oncology, University Hospital Halle (Saale),
Ernst-Grube-Str. 40, 06120, Halle (Saale), Germany.
Telephone no: +49-345-557-3453/4027
Email: Daniel.Medenwald@uk-halle.de

Appendix 2: Missing Treatment information: Sensitivity Analysis

Table 1. Characteristics of patients diagnosed with endometrial cancer 2004-2014 according to socioeconomic deprivation quintiles (missing treatment was considered as "not received").

		Deprivation Level				
	All patients	**Least Deprived**	**2**	**3**	**4**	**Most Deprived**
Number of patients	31596	3971	6019	5544	7072	8990
Alive at end of follow-up (%)	22141 (70.1)	2870 (72.3)	4304 (71.5)	3846 (69.4)	4917 (69.5)	6204 (69.0)
Mean age at diagnosis **(SD)**	67.7 (11.3)	67.0 (11.4)	67.1 (11.5)	67.2 (11.6)	68.1 (11.2)	68.2 (11.1)
**Period of Diagnosis (%)**						
2004-2008	11943 (37.8)	1472 (37.1)	2066 (34.3)	1739 (31.4)	2752 (38.9)	3914 (43.5)
2009-2013	19653 (62.2)	2499 (62.9)	3953 (65.7)	3805 (68.6)	4320 (61.1)	5076 (56.5)
**Type (%)**						
Low grade	24982 (79.1)	3046 (76.7)	4668 (77.6)	4312 (77.8)	5689 (80.4)	7267 (80.8)
High grade	6614 (20.9)	925 (23.3)	1351 (22.4)	1232 (22.2)	1383 (19.6)	1723 (19.2)
**Grade (%)**						
I	11623 (36.8)	1234 (31.1)	1997 (33.2)	2021 (36.5)	2674 (37.8)	3697 (41.1)
II	13658 (43.2)	1855 (46.7)	2724 (45.3)	2331 (42.0)	3096 (43.8)	3652 (40.6)
III	6315 (20.0)	882 (22.2)	1298 (21.6)	1192 (21.5)	1302 (18.4)	1641 (18.3)
**Stage at Diagnosis (%)**						
I	13269 (42.0)	1338 (33.7)	2182 (36.3)	1668 (30.1)	3370 (47.7)	4711 (52.4)
II	1410 (4.5)	158 (4.0)	227 (3.8)	186 (3.4)	327 (4.6)	512 (5.7)
III	1816 (5.7)	231 (5.8)	366 (6.1)	254 (4.6)	394 (5.6)	571 (6.4)
IV	726 (2.3)	109 (2.7)	149 (2.5)	113 (2.0)	151 (2.1)	204 (2.3)
Missing	14375 (45.5)	2135 (53.8)	3095 (51.4)	3323 (59.9)	2830 (40.0)	2992 (33.3)
**Treatment (%)**						
Radiotherapy	9744 (30.8)	1010 (25.4)	1609 (26.7)	1242 (22.4)	2477 (35.0)	3406 (37.9)
Chemotherapy	1290 (4.1)	192 (4.8)	291 (4.8)	231 (4.2)	223 (3.2)	353 (3.9)
Surgery	23446 (74.2)	2518 (63.4)	4146 (68.9)	3492 (63.0)	5548 (78.5)	7742 (86.1)

Abbreviations: SD= Standard Deviation

Table 2: Cox proportional hazards model survival estimates according to deprivation levels of patients diagnosed with endometrial cancer in Germany, 2004–2014 (missing treatment was considered as "not received").

	N of Events	Hazard Ratios (95%CI)				
		Model 1	Model 2	Model 3	Model 4	Model 5
**All Stages**	3446					
Q1		1.00 (ref)	1.00 (ref)	1.00 (ref)	1.00 (ref)	1.00 (ref)
Q2		0.91 (0.80-1.04)	0.93 (0.82-1.06)	0.93 (0.82-1.06)	0.94 (0.82-1.06)	0.92 (0.80-1.04)
Q3		1.00 (0.87-1.15)	1.01 (0.88-1.16)	1.02 (0.89-1.17)	1.02 (0.89-1.17)	0.99 (0.85-1.15)
Q4		0.85 (0.75-0.96)	0.92 (0.81-1.04)	1.00 (0.89-1.13)	1.03 (0.91-1.16)	0.99 (0.84-1.17)
Q5		0.93 (0.83-1.04)	1.02 (0.91-1.14)	1.06 (0.95-1.19)	1.10 (0.98-1.23)	1.06 (0.89-1.25)
**Stage I**	1867					
Q1		1.00 (ref)	1.00 (ref)		1.00 (ref)	1.00 (ref)
Q2		0.99 (0.82-1.20)	1.00 (0.83-1.22)		1.02 (0.84-1.22)	0.98 (0.80-1.19)
Q3		1.23 (1.00-1.51)	1.22 (0.99-1.49)		1.21 (0.99-1.48)	1.12 (0.90-1.40)
Q4		1.06 (0.89-1.26)	1.09 (0.92-1.30)		1.13 (0.95-1.35)	1.03 (0.82-1.30)
Q5		1.19 (1.01-1.41)	1.25 (1.05-1.48)		1.30 (1.09-1.53)	1.24 (0.98-1.57)
**Stage II**	373					
Q1		1.00 (ref)	1.00 (ref)		1.00 (ref)	1.00 (ref)
Q2		0.67 (0.44-1.02)	0.67 (0.44-1.02)		0.68 (0.45-1.04)	0.65 (0.43-1.00)
Q3		0.90 (0.59-1.35)	0.93 (0.62-1.41)		0.93 (0.62-1.42)	1.00 (0.63-1.58)
Q4		0.89 (0.62-1.28)	0.93 (0.65-1.33)		0.97 (0.67-1.39)	0.87 (0.53-1.42)
Q5		0.88 (0.63-1.24)	0.92 (0.66-1.29)		0.97 (0.69-1.36)	0.88 (0.52-1.48)
**Stage III**	750					
Q1		1.00 (ref)	1.00 (ref)		1.00 (ref)	1.00 (ref)
Q2		0.92 (0.71-1.18)	0.94 (0.73-1.21)		0.92 (0.72-1.19)	0.91 (0.70-1.18)
Q3		0.84 (0.63-1.11)	0.91 (0.69-1.21)		0.88 (0.66-1.17)	0.83 (0.61-1.13)
Q4		0.97 (0.76-1.24)	1.04 (0.81-1.33)		1.03 (0.80-1.32)	0.92 (0.66-1.28)
Q5		0.90 (0.71-1.14)	0.94 (0.74-1.19)		0.95 (0.75-1.21)	0.79 (0.55-1.12)
**Stage IV**	456					
Q1		1.00 (ref)	1.00 (ref)		1.00 (ref)	1.00 (ref)
Q2		1.00 (0.74-1.35)	0.96 (0.71-1.30)		0.96 (0.71-1.31)	0.98 (0.72-1.34)
Q3		0.97 (0.70-1.34)	0.92 (0.66-1.28)		0.91 (0.66-1.28)	0.98 (0.68-1.40)
Q4		0.83 (0.60-1.14)	0.80 (0.58-1.11)		0.81 (0.58-1.12)	0.97 (0.64-1.49)
Q5		0.90 (0.68-1.20)	0.88 (0.66-1.17)		0.91 (0.68-1.22)	1.02 (0.67-1.57)

Model 1: Adjusted for age and year of diagnosis. **Model 2**: Same as Model 1 plus Grade and Type. **Model 3**: Same as Model 2 plus stage Model 4: Same as Model 3 plus treatment. Stratified analysis: Same as Model 2 plus treatment, Model 5: Same as Model 4 plus registry. Stratified analysis: Same as Model 2 plus treatment and registry

Abbreviations: Q= Quintiles, CI= Confidence Interval.
