# Supplementary material for: Socioeconomic disparities in endometrial cancer survival in Germany: a survival analysis using population-based cancer registry data
Source: J Cancer Res Clin Oncol. 2022 Jan 22;148(5):1087–95. doi: 10.1007/s00432-021-03908-9 (PMC9015991; doi:10.1007/s00432-021-03908-9)
Supplement: Supplementary file 3 — Supplementary file3 (PDF 300 KB) [file 432_2021_3908_MOESM3_ESM.pdf]

Socioeconomic disparities in Endometrial Cancer survival in Germany: A survival analysis using population-based cancer registry data.

Bedir, Ahmed[1]; Abera, Semaw Ferede[1]; Vordermark, Dirk[1,2]; Medenwald, Daniel[1,2]

1. Department of Radiation Oncology, Health Services Research Group, University Hospital Halle (Saale), Ernst-Grube-Str. 40, 06120, Halle (Saale), Germany.
2. Department of Radiation Oncology, University Hospital Halle (Saale), Ernst-Grube-Str. 40, 06120, Halle (Saale), Germany.

Acknowledgments: None.

Address for correspondence:
Daniel Medenwald
Department of Radiation Oncology, University Hospital Halle (Saale),
Ernst-Grube-Str. 40, 06120, Halle (Saale), Germany.
Telephone no: +49-345-557-3453/4027
Email: Daniel.Medenwald@uk-halle.de

Appendix 3: R Code.

1- Kaplan Meier Curve plots.

```
KM <- survfit(Surv(finaldate, TOD10==1) ~ gisdqd, type="kaplan-meier", data=endsccses)
fit <- surv_fit(Surv(endsccses$finaldate,endsccses$TOD10)~1, data=endsccses)
ggsurvplot(KM, xlim = c(0, 10),ylim =c(0.25, 1), data = endsccses,
        legend.labs = c("Least Deprived (Q1)","Q2","Q3","Q4","Most Deprived (Q5)")
)+xlab("Time (years)")
```

2- Kaplan Meier estimates.

```
summary(survfit(Surv(finaldate,TOD10==1) ~gisdqd, data = endsccses), times = 5)
```

3- Cox regression models for all stages adjusted for all variables.

```
x<-coxph(Surv(time = endsccses$finaldate, event = endsccses$TOD10==1)~
gisdqd+age+diagyear+typ+GRAD+stage_dx+PTO+PTB+PTC+GKZbl, data=endsccses)

exp(cbind("Hazard Ratio" = coef(x), confint.default(x, level = 0.95)))
```

4- Cox regression models stratified according to stage, adjusted for all variables.

```
endsessub<-endsccses %>% filter(stage_dx%in%c("1"))
surv_object2 <- Surv(time = endsessub$finaldate, event = endsessub$TOD10==1)
res.cox <- coxph(surv_object2~gisdqd+age+diagyear+GRAD+typ+PTO+PTB+PTC+GKZbl, data =
endsessub)
exp(cbind("Hazard Ratio" = coef(res.cox), confint.default(res.cox, level = 0.95)))
```
